# Tumour–host interactions in *Drosophila*: mechanisms in the tumour micro‐ and macroenvironment

**DOI:** 10.1002/1878-0261.70207

**Published:** 2026-01-17

**Authors:** José Teles‐Reis, Tor Erik Rusten

**Affiliations:** ^1^ Center for Cancer Cell Reprogramming, Institute of Clinical Medicine, Faculty of Medicine University of Oslo Norway; ^2^ Department of Molecular Cell Biology, Institute for Cancer Research Oslo University Hospital Norway

**Keywords:** cachexia, cell competition, *Drosophila*, tumour macroenvironment, tumour microenvironment, tumour–host interactions

## Abstract

Traditionally, cancer has been viewed largely as a disease of the cell, with extensive research centred on how mutations in driver genes trigger cellular transformation. Beyond cell‐intrinsic changes, cancer unfolds as a systemic disease driven by an intricate dialogue between malignant cells and the host's organs and tissues. Modelling this multilayered phenomenon is challenging, as it requires recapitulating coordinated interactions within and across multiple organs, inside an organism that is contended with maintaining normal physiology. In recent years, *Drosophila melanogaster* has emerged as a powerful model for revealing fundamental mechanisms by which the tumour and host mutually interact. In this review, we highlight recent findings that unravel the intricacies of tumour–host biology using *Drosophila*. At the microenvironment level, we synthesise mechanistic findings on how tumour growth is modulated through interactions with neighbouring tumour subclones, nonmutated wild‐type cells and the immune system. At the macroenvironment level, work in *Drosophila* has provided mechanistic insights into how tumourigenesis causes systemic host health degeneration and accelerates death, collectively termed paraneoplastic effects. Tumours can remotely induce systemic metabolic rewiring and cachectic tissue wasting to promote progression, while simultaneously compromising the function of several tissues, such as the renal system, blood–brain barrier, the gut and blood haemostasis. Additionally, we discuss how the microbiota and sexual dimorphism have been shown to affect the tumour–host interplay. With this review, we synthesise recent advances in *Drosophila* tumour–host biology and illustrate how this model illuminates cancer's systemic nature.

## Graphical abstract

This review examines how tumour–host crosstalk takes place at multiple levels of biological organisation, from local cell competition and immune crosstalk to organism‐wide metabolic and physiological collapse. Here, we integrate findings from Drosophila melanogaster studies that reveal conserved mechanisms through which tumours hijack host systems to drive growth and lethality.

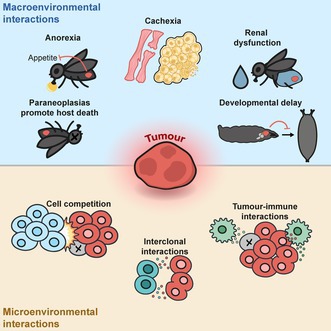

**Abbreviations**
AiP: apoptosis‐induced proliferation
AKH: adipokinetic hormone
AMPs: antimicrobial peptides
APC: Adenomatous polyposis coli
BBB: blood–brain barrier
bZip: basic leucine Zipper
CA: corpus allatum
CC: corpus cardiacum
cGMP: cyclic guanosine monophosphate
CNS: central nervous system
DAMPs: damage‐associated molecular patterns
Dcp‐1: Death caspase‐1
Diap1: Death‐associated inhibitor of apoptosis 1
Dilps: *Drosophila* insulin‐like peptides
Dpp: Decapentaplegic
Drice: Death‐related ICE‐like caspase
Dronc: Death regulator Nedd2‐like caspase
Dsx: Doublesex
Duox: dual oxidase
EAD: eye‐antennal disc
EBs: enteroblast cells
ECM: extracellular matrix
ECs: enterocytes
EDAC: epithelial defence against cancer
EEs: enteroendocrine cells
EGFR: epidermal growth factor receptor
Egr: Eiger
eIF2α: Eukaryotic translation initiation factor 2 subunit 1
EMT: epithelial‐to‐mesenchymal transition
ER: endoplasmic reticulum
ESCRT: endosomal sorting complexes required for transport
Fru: Fruitless
gbb: Glass bottom boat
GOF: gain‐of‐function
GPCR: G‐protein‐coupled receptor
H_2_O_2_
: hydrogen peroxide
HIF‐1α: Hypoxia‐inducible factor 1/Sima
Hml: Hemolectin
IGFBPs: insulin growth factor‐binding proteins
Imd: immune deficiency pathway
InR: Insulin receptor
IPCs: insulin‐producing cells
Ire1: Inositol‐requiring enzyme‐1
ISCs: intestinal stem cells
ITP_F_
: ion transport peptide (isoform F)
JAK–STAT: Janus kinase‐Signal transducer and activator of transcription proteins pathway
JhI‐21: Juvenile hormone Inducible‐21
JNK: c‐Jun N‐terminal kinase pathway
l(2)gl: lethal (2) giant larvae
Ldh: Lactate Dehydrogenase
Lgr3: Leucine‐rich repeat‐containing G protein‐coupled receptor 3
LOH: loss of heterozygosity
MAPK: Mitogen‐activated protein kinase
MARCM: Mosaic Analysis with a Repressible Cell Marker
MCT1: Monocarboxylate transporter 1
Mmps: matrix metalloproteinases
modSP: modular serine protease
mTOR: mechanistic target of rapamycin
NMDAR: N‐methyl‐d‐aspartate receptor
NOS: nitric oxide synthase
NPF: neuropeptide F
NUCB1: nucleobinding 1
Onc: oncogene
PAMPs: pathogen‐associated molecular patterns
Path: Pathetic
PDH: pyruvate dehydrogenase
PDK: pyruvate dehydrogenase kinase
Pepck1: phosphoenolpyruvate carboxykinase 1
PERK: protein kinase R‐like endoplasmic reticulum kinase
PG: prothoracic gland
Phf7: PHD finger protein 7
PI3K: phosphatidylinositol 3‐kinase
PPOs: pro‐phenoloxidases
PS: phosphatidylserine
Pvf1: PDGF‐ and VEGF‐related factor 1
Ras^V12^
: oncogenic Ras
RHG: Reaper, Hid, Grim
ROS: reactive oxygen species
SASP: senescence‐associated secretory phenotype
scrib: scribble
slif: slimfast
SPE: Spatzle‐Processing Enzyme
SPG: subperineurial glia
spz: spatzle
Sxl: Sex‐lethal
TAG: triacylglycerol
TCA: tricarboxylic acid cycle
Tg: Transglutaminase
TGFβ: Transforming growth factor beta
TIMP: tissue inhibitor of metalloproteinase
TkR99D: Tachykinin‐like receptor at 99D
TNFα: Tumour Necrosis Factor alpha
Tra: Transformer
TSG: tumour suppressor gene
TSG101: tumour susceptibility gene 101
upd: unpaired
UPR: unfolded protein response
WD: wing imaginal disc
wg: wingless
WT: wild‐type
Xbp1: X box binding protein‐1
yki^3SA^
: oncogenic yorkie
zfh1: Zinc finger homeodomain 1

## 1 Introduction

Tumour development does not occur in isolation but rather unfolds within the dynamic and responsive environment of host tissues. The complexity of the tumour–host interface becomes immediately evident upon microscope observation of patient biopsy samples. These typically reveal not only malignant cells but also a diverse array of host cell types, including untransformed homotypic cells (cells of the same type as the tumour's cell of origin) and heterotypic cells (different cell types such as fibroblasts, endothelial cells and immune cells), alongside aberrant extracellular matrix deposition. The emergence of transformed cells has been shown to reshape the localization, density, and signalling behaviour of surrounding host cells, which respond actively and can engage in direct interactions with cancer cells. This complexity is commonly referred to as the tumour microenvironment and it is widely recognised as a key modifier of tumour progression and therapy response [1].

Beyond these local interactions, tumours also drive pathological changes in distant host tissues. These systemic effects, known as paraneoplastic syndromes [2, 3, 4, 5, 6], include a wide spectrum of conditions, such as tissue wasting (cachexia), anorectic feeding behaviour, endocrine, neuronal and haematological alterations, renal dysfunction and oedema. The distant host tissues that are affected by, and interact with, the primary tumour, also known as the tumour macroenvironment, are believed to be influenced by tumour‐secreted factors that enter the circulation. Some paraneoplastic syndromes are highly prevalent and can significantly impact disease outcome and treatment. For instance, cancer cachexia, characterised by the wasting of systemic tissues, is estimated to affect approximately 33% of cancer patients, preclude chemotherapy intervention [7] and is strongly associated with reduced overall survival [8].

The current dominant cancer therapeutic paradigm remains focussed on directly targeting tumour cells with radiotherapy, chemotherapy or targeted drugs. However, the clinical success of immunotherapy, such as with immune checkpoint blockade and adoptive cell transfer, has proven the powerful therapeutic potential of strategies that engage and reprogram host tissues to mount an antitumour response [9]. Despite growing recognition of the active role that host tissues play in shaping cancer progression, our understanding of the basic mechanisms underlying tumour–host crosstalk remains limited. This in turn limits the development of novel therapies aimed at modulating tumour‐induced host responses.

Studying tumour–host biology is inherently challenging, as it is a phenomenon that involves complex interactions between multiple cell types and organs. It therefore requires experimental models that not only capture tissue and systems level complexity but also allow for genetic manipulation of host tissues independently of tumour induction, in order to dissect host gene function during tumourigenesis. The fruit fly, *Drosophila melanogaster*, has emerged as a powerful system for uncovering conserved core principles behind tumour–host interplay. Flies provide a miniaturised and highly scalable *in vivo* model that combines multi‐organ complexity with exceptionally tractable genetics. In this work, we summarise the recent findings in the field of tumour–host biology from studies harnessing the powerful *Drosophila* model system.

## 2 Getting to know the host: a brief view on the anatomy and physiology of *Drosophila*

**Fig. 1:**
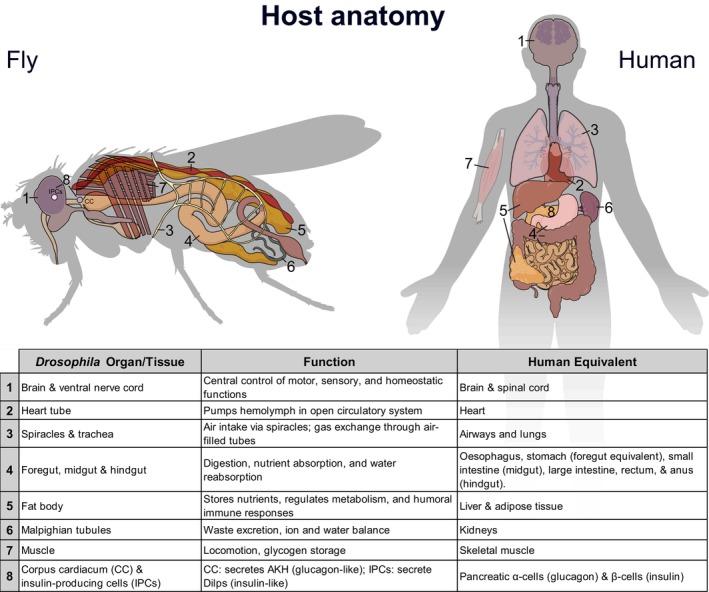
Anatomical and functional parallels between the fly and human organs. Schematic representation of the major organs in *Drosophila* and their human equivalents. Organs are labelled 1–8, with corresponding function and human parallels summarised in the accompanying table below.

Most human tissues and organs have structural and/or functional counterparts in *Drosophila* (Fig. 1). Flies have an open circulatory system and a dorsally located heart tube, which pumps insect blood (hemolymph) to deliver nutrients throughout the body [10]. In insects, gas exchange to support aerobic respiration is achieved through the tracheal system, a highly branched network of hollow tubes that allows air from the exterior to circulate inside the body. Air enters the trachea through small openings in the cuticle called spiracles [11]. It was previously believed that, in contrast to humans, insects did not require circulating cells to transport oxygen and that gas exchange occurred solely via diffusion. However, recent work has shown that myeloid‐like cells in *Drosophila* can transport oxygen and provide significant respiratory support [12].

Flies possess a highly complex central nervous system (CNS), which is subdivided into the brain and ventral nerve chord, which governs nearly all aspects of their biology, including motor control, sensory processing, memory, sleep, reproduction and physiological homeostasis. Within the CNS are several groups of neurons that produce neuropeptides and hormones, which can act locally or be secreted into circulation to exert systemic effects. A central population of neuroendocrine cells are the insulin‐producing cells (IPCs), which express and secrete the *Drosophila* insulin‐like peptides (Dilps) 2, 3 and 5. Circulating Dilps bind the insulin receptor in the different tissues to stimulate anabolic processes coordinated with nutrient availability. The IPCs are thus functionally analogous to the human pancreatic β‐cells [13]. Another key hormone‐secreting organ, located adjacent to the brain, is the ring gland. The larval ring gland is subdivided into three endocrine regions that produce hormones that regulate development and metabolism: the prothoracic gland (PG), the corpus allatum (CA) and the corpus cardiacum (CC). In adulthood, the prothoracic gland degenerates. The CC shows functional similarity to the pancreatic α‐cells, as it produces adipokinetic hormone (AKH), a functional analogue to human glucagon. AKH functions in opposition to insulin during nutrient deficiency to promote carbohydrate mobilisation and lipolysis for energy generation [14].

The major energy storage organ in *Drosophila* is the fat body, an adipose‐ and liver‐like tissue responsible for storing both carbohydrates and lipids. It acts as a central nutrient sensor and plays a critical role in maintaining energy homeostasis by responding to hormonal signals such as Dilps and AKH. As in humans, excess sugar is stored in the fat body as glycogen, a branched polysaccharide composed of glucose units [15], and also converted into fatty acids and stored as triacylglycerol (TAG) in lipid droplets [16]. However, unlike in mammals, the main circulating sugar in *Drosophila* is trehalose, a disaccharide composed of two glucose molecules [17]. Another storage location of glycogen is the muscle. Flies possess a highly organised muscle system that closely resembles skeletal muscles of humans. *Drosophila* somatic muscles are composed of multinucleated fibres, and movement is powered by sarcomeres [18].

The fly gut is highly regionalized along the anterior–posterior axis in terms of tissue structure and gene expression, enabling the execution of regionalized digestive functions. It is divided into three anatomical regions: foregut, midgut and hindgut. The gut is composed of several cell types: intestinal stem cells (ISCs); nutrient‐absorptive enterocytes (ECs); hormone‐secreting enteroendocrine cells (EEs) that regulate systemic physiology; immature enteroblast cells (EBs), which differentiate into ECs or EBs, enteric neurons and visceral muscle. Like in mammals, the gut is highly plastic and can dynamically adapt to several physiological and stress stimuli, including reproduction status, nutrient availability, infection, inflammation and injury [19].

Connected to the midgut are the Malpighian tubules, which serve as the insect's excretory organs, alike the kidney. They filter waste products from the hemolymph and generate urine within the tubule lumen. The tubules are composed of two main cell types, the principal cells and the stellate cells. Secretion of potassium and chloride ions into the lumen of the tubule by transporters in principal cells and stellate cells, respectively, drives the osmotic movement of water. Toxic solutes are simultaneously excreted to the lumen through active transport systems [20]. Animal water balance is tightly regulated in the Malpighian tubules by hormones that can signal to principal and stellate cells to regulate ion movement, and thus osmotic pressure [20]. Finally, the ions and water used for excretion can be later reabsorbed in the hindgut, a process that is similarly tightly regulated by hormones according to animal needs [20].

Lastly, *Drosophila* is highly competent at resisting and combating pathogens. Their immunity defences range from behavioural (avoidance of pathogen laced food) to cellular and humoral immunity [21]. Unlike vertebrates, flies lack an adaptive immune system but possess remarkably effective innate immunity. The main immune‐responsive tissues are the haemocytes (myeloid‐like circulating immune cells) and the fat body (liver/adipose tissue). A key component of *Drosophila* innate immunity is the Toll signalling pathway, which was first characterised in flies. This pathway is essential for recognising pathogen‐associated molecular patterns (PAMPs) and inducing the expression of various immune effectors, most notably the antimicrobial peptides (AMPs) [21]. Haemocytes are functionally subdivided into three cell subtypes: the plasmatocytes, macrophage‐like cells that can engulf bacteria and cellular debris; crystal cells, which can mount a melanisation response (formation of fibrin‐like scabs) and aid in oxygen transport [12]; and lamellocytes, which can encapsulate larger pathogens [21].

Together, this section highlights the remarkable systemic complexity of the *Drosophila* model and its parallels with human physiology, making it a valuable system for exploring tumour–host interactions.

## 3 Putting tumours into context: Deciphering tumour–host biology with *Drosophila*

*Drosophila* has proven to be an excellent model for cancer research by enabling foundational discoveries and faithfully recapitulating key features of human malignancies. Seminal studies in fruit flies have uncovered fundamental cellular principles underlying several cancer hallmarks such as cell cycle regulation [22, 23], intercellular signalling [24], cell migration [25] and apoptosis [26, 27, 28, 29]. Moreover, fly research has greatly advanced our understanding of cellular transformation by identifying numerous oncogenes (Onc) and tumour suppressor genes (TSGs), and revealing how their mutation alters cell behaviour [30, 31, 32, 33, 34, 35, 36, 37, 38, 39, 40, 41].

Advanced genetic methods in flies, such as binary expression systems and Mosaic Analysis with a Repressible Cell Marker (MARCM), enable the study of tumours of different origin sites by allowing the introduction of oncogenic insults into restricted cell populations within any tissue of interest. Importantly, they also allow for precise cell genetic manipulation, enabling the assessment of autonomous gene function during tumour development (methods for modelling tumourigenesis in flies reviewed in [42]). Common sites for tumour initiation in *Drosophila* include epithelia, such as the larval eye‐antennal disc (EAD) and wing imaginal disc (WD), which are larval epithelial structures that give rise to adult tissues [43]; the adult ovarian epithelial layer; stem cell populations in the larval CNS or adult gut; and the haematopoietic cell lineages.

Besides shedding light on the internal mechanisms of tumourigenesis, more recent work has begun to unravel in flies how tumours and host tissue interact to shape disease progression. Here, we will examine key evidence that reveals mechanisms operating at both the micro‐ and macroenvironmental levels.

## 4 Microenvironmental interactions

### 4.1 Interactions between tumours and the neighbouring epithelial tissue

#### 4.1.1 The role of cell competition in tumour progression

The process of cell competition was first observed in *Drosophila* through the study of heterozygous mutants for ribosomal protein genes, known as Minute. Although Minute animals develop more slowly, their organs form correctly. However, when Minute cell clones are surrounded by wild‐type (WT) cells, they are eliminated from the tissue [44]. Their removal was later shown to specifically occur through apoptosis at the interface with the healthy neighbours [45]. Since this initial discovery, numerous other cellular insults have been shown to trigger cell competition. This includes aneuploidy [46], proteotoxic stress [47], oxidative stress signalling [48], mechanical constraints [49] and mitochondrial defects [50], collectively demonstrating that cell competition is a central mechanism for maintaining tissue homeostasis by removing less fit cells.

**Fig. 2:**
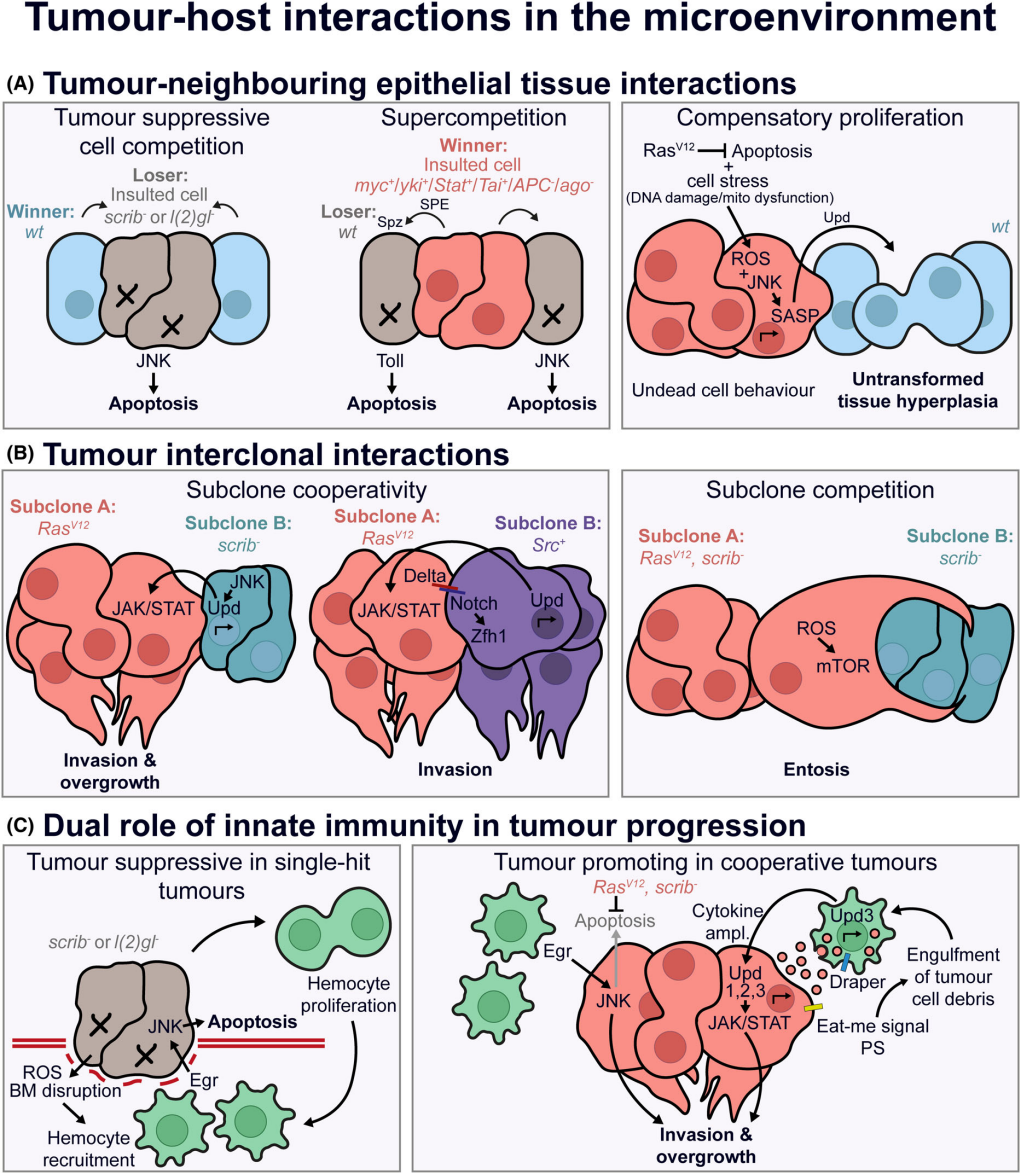
The interplay of epithelial, tumour subclones, and immune cell interactions in the tumour microenvironment. Illustrations summarising the main processes occurring within the tumour microenvironment. (A) At the tumour‐neighbouring epithelial level, cell competition can act as either a tumour‐suppressive or a tumour‐promoting mechanism (supercompetition), depending on the nature of the genetic insults in the tissue. In addition, transformed cells can drive overgrowth of adjacent wildtype tissue through aberrant activation of regenerative responses. (B) Tumour subclones with distinct mutational identities can influence each other's fate. Cooperative interactions can enhance malignant properties between neighbouring subclones, whereas competitive interactions can result in the elimination of one clone by another in its vicinity. (C) Tumours can recruit innate immune cells through reactive oxygen species (ROS) production and basement membrane (BM) disruption. Haemocytes can promote tumour cell elimination via TNFα (egr) signalling, but acquisition of cooperative mutations can shift their activity from anti‐tumourigenic to pro‐tumourigenic. Furthermore, engulfment of tumour cell debris can amplify cytokine signalling, promoting tumour growth.

Evidence that cell competition influences cancer initiation first emerged from studies of clones undergoing loss of heterozygosity (LOH) for TSGs involved in cell polarity, such as *scribble* (scrib) and *lethal (2) giant larvae* (l(2)gl) (Fig. 2A, Table 1). The behaviour of tumour clones with polarity loss mirrors that of Minute clones. When all cells in the tissue are mutant for *scrib* or *l(2)gl*, they are retained and can grow into a large tumour. In contrast, when polarity mutant clones are surrounded by WT cells, they are eliminated through apoptosis [51, 52, 53]. Tumour‐suppressive cell competition driven by WT cells has also been observed following the spontaneous gain‐of‐function (GOF) of several oncogenes, including *Ras* [54], and identified in mammalian studies, *Src* [55] and *Erbb2* [56]. However, the elimination of these oncogene‐transformed cells seems to occur through a distinct mode of cell competition, in which the transformed cells are removed from the epithelial layer via extrusion promoted by the neighbouring WT cells, a process that is termed epithelial defence against cancer (EDAC) [54, 57].

**Table 1:** Drivers of tumour microenvironmental interactions in *Drosophila*.

Loser	Winner	Human orthologue	Ref.
**Cell competition**			
*scrib* ^*−*^	*wt* epithelial neighbours	*SCRIB*	[51, 52]
*l(2)gl* ^*−*^	*wt* epithelial neighbours	*LLGL1/2*	[53]
*Ras* ^*V12*^	*wt* epithelial neighbours	*RAS* family	[54]
**Supercompetition**			
*wt* epithelial neighbours	*myc* ^*GOF*^	*MYC*	[41, 58]
*wt* epithelial neighbours	*yki* ^*GOF*^	*YAP1*	[59]
*wt* epithelial neighbours	*ft* ^*−*^	*FAT4*	[60]
*wt* epithelial neighbours	*Hop* ^*GOF*^	*JAK* family	[61]
*wt* epithelial neighbours	*Tai* ^*GOF*^	*NCOA* family	[62]
*wt* epithelial neighbours	*Apc* ^*−*^	*APC*	[63, 64]
*wt* epithelial neighbours	*Axn* ^*−*^	*AXIN1/2*	[63]
*wt* epithelial neighbours	*ago* ^*−*^	*FBXW7*	[65]

Induction of nonautonomous hyperplasia			
Mutant cells	Overproliferative neighbours	Human orthologue	Ref.
*raf* ^*GOF*^ + *hep* ^*GOF*^	*wt* epithelial neighbours	*RAF* family + *MAP2K7*	[66]
*ptc* ^*−*^ + *Dark* ^*−*^	*wt* epithelial neighbours	*PTCH1/2* + *APAF1*	[67]
*vps25* ^*−*^	*wt* epithelial neighbours	*VPS25*	[68, 69]
*ept* ^*−*^	*wt* epithelial neighbours	*TSG101*	[70]
*Rab5* ^*−*^	*wt* epithelial neighbours	*RAB5*	[71]
*Ras* ^*V12*^ + cell stress (DNA damage and mito dysfunction)	*wt* epithelial neighbours	*RAS* family	[72, 73, 74]
*EGFR* ^*GOF*^ *+ psq* ^*−*^	*wt* mesenchymal neighbours	*EGFR* + ?	[75, 76]

Tumour interclonal interactions			
Subclone A	Subclone B	Human orthologue	Ref.
**Cooperativity**			
*Ras* ^*V12*^	*scrib* ^*−*^	*RAS* family//*SCRIB*	[77]
*Ras* ^*V12*^	*Src* ^*GOF*^	*RAS* family//*SRC* family	[78]
**Competition**			
*Ras* ^*V12*^, *scrib* ^*−*^	*scrib* ^*−*^	*RAS* family, *SCRIB*//*SCRIB*	[79]

Tumour cells are known to exploit endogenous processes to promote malignancy, including developmental programmes such as epithelial‐to‐mesenchymal transition (EMT) and regenerative responses such as pro‐proliferative inflammation. Notably, studies in *Drosophila* have shown that transformed cells can also hijack cell competition to eliminate neighbouring untransformed WT cells. This reversed dynamic, where the genetically insulted cell gains the capacity to outcompete and eliminate its neighbours, is termed supercompetition and has been observed following mutations of several Onc and TSGs (Fig. 2A, Table 1). Supercompetitive cells are seen upon elevation of Myc [41, 58], yorkie activity [59, 60], Janus kinase‐Signal transducer and activator of transcription proteins (JAK–STAT) signalling [61], Taiman [62] and loss of *Adenomatous polyposis coli* (APC) [63, 64], *Axin* [63], or *archipelago* (ago) [65]. Importantly, blocking the supercompetitive elimination of neighbouring WT cells has been shown to reduce tumour growth in *fat* mutant carcinomas [60] and *APC*‐deficient adenomas [64]. Strikingly, it has been later demonstrated that *APC* mutant cells can also act as supercompetitors in the mammalian gut [80, 81, 82]. How the death of surrounding cells contributes to tumour growth remains to be elucidated. It is possible that it creates additional space for expansion, increases nutrient availability in the microenvironment, or triggers wound‐like inflammatory programmes that stimulate tumour proliferation.

A key area of investigation concerns the signalling mechanisms through which cells are identified as ‘losers’ and targeted for elimination. Activation of the c‐Jun N‐terminal kinase (JNK) stress pathway appears to be a consensus downstream event leading to the elimination of the unfit cells across various cell competition scenarios [41, 48, 64, 83, 84] (Fig. 2A). Differences between winners and losers on the uptake of trophic factors, such as the survival‐promoting Decapentaplegic (Dpp) ligand, have been proposed to modulate cell competition [45]. In addition, innate immunity pathways play crucial roles in both Minute cell competition and Myc‐driven supercompetition [60, 62, 85, 86] (Fig. 2A). Myc winners upregulate the Toll ligand *spatzle* (spz), and genes involved in its activation *Spatzle‐Processing Enzyme* (SPE) and *modular serine protease* (modSP) to drive the death of neighbouring loser clones through Toll signalling. Loss of *SPE* in *myc* overexpressing cells reduces the elimination of the surrounding WT cells [86].

Proteostasis status has emerged as another key determinant of cellular fitness in cell competition [47, 87, 88, 89, 90]. The unfolded protein response (UPR), triggered by endoplasmic reticulum (ER) stress, becomes activated in loser cells. This includes the activation of the two major branches of the UPR: the protein kinase R‐like endoplasmic reticulum kinase (PERK)‐Eukaryotic translation initiation factor 2 subunit 1 (eIF2α) [87, 88] and the Inositol‐requiring enzyme‐1 (Ire1)‐X box binding protein‐1 (Xbp1) [88, 90] signalling axes. Theoretically, using the proteostasis state as a sensor to identify unfit cells is logical, as a wide range of stressors can disrupt protein folding, and many cancer types exhibit activation of UPR pathways. Downregulation of PERK prevents the accumulation of p‐eIF2α in loser cells and reduces their elimination [88]. Mechanistically, the PERK‐eIF2α pathway forms a feed‐forward loop with the basic leucine Zipper (bZip) transcription factor Xrp1 to promote loser cell elimination [87, 88, 89]. In the context of cancer, disruption of the Ire1‐Xbp1 has been shown to enhance the tumour‐suppressive elimination of *scrib* mutant cells. However, when this pathway is disrupted in the neighbouring WT cells, the elimination of *scrib* mutant cells is suppressed, suggesting that the relative proteostasis status determines winner versus loser identity [90]. This concept of relative fitness is a hallmark of cell competition, as originally demonstrated in the case of Myc winners, where differences in Myc levels tip the competitive outcomes [41].

Metabolic alterations also contribute to the competitive dynamics. Myc elevated cells specifically rewire their metabolism when facing WT neighbours to increase sugar uptake and glycolysis, which enhances their fitness. This behaviour was found to be dependent on p53, suggesting that although p53 is widely mutated in cancer, it may be critical during tumour initiation for initial clone expansion [91]. How p53 senses the heterotypic confrontation with wild‐type neighbours to trigger this metabolic reprogramming still remains an open question. Furthermore, glutamate signalling was recently shown to be a critical mediator of cell competition through metabolic remodelling. Cells lacking a subunit of the glutamate receptor, N‐methyl‐d‐aspartate receptor (NMDAR), are eliminated only when neighboured by WT cells [84]. Similarly, loser behaviour is triggered by autonomous inhibition of glutamate synthesis or transport [92]. Mechanistically, lower glutamate signalling leads to a JNK and pyruvate dehydrogenase kinase (PDK)‐mediated inactivation of pyruvate dehydrogenase (PDH), which is required for production of acetyl‐CoA for the TCA cycle. Lactate dehydrogenase (Ldh) then converts pyruvate to lactate, which is exported through the monocarboxylate transporter 1 (MCT1) to neighbouring cells. Strikingly, *myc*‐overexpressing cells upregulate both an NMDAR subunit and exhibit increased glutamate levels. Depletion of glutamate signalling, transport or synthesis in *myc* overexpressing cells prevents supercompetition [84, 92]. Loser cells were found to shuttle lactate into Myc winner cells via MCT1, which is required for supercompetition [84]. Future studies should clarify how increased glycolytic flux and lactate uptake promote competitiveness.

#### 4.1.2 Cancer cells exploit the regenerative compensatory proliferation program and trigger the abnormal growth of adjacent untransformed tissue

Cell death resulting from injury or other stress conditions requires tissues to regenerate lost cells to maintain structure and function through compensatory proliferation. Studies in flies have shown that the machinery driving cell death is intimately linked with regenerative programmes [93, 94, 95]. Controlled cell death (apoptosis) is an evolutionarily conserved and tightly regulated process, orchestrated by the serial activation of cysteine proteases known as caspases [96]. Caspases are divided into two functional groups: initiator caspases that sense pro‐apoptotic signals and initiate the caspase cascade; and effector caspases, which, once activated by the initiator caspases, cleave several cellular substrates to execute apoptosis. In flies, the main initiator caspase is the death regulator Nedd2‐like caspase (Dronc), while the primary effector caspases are Death‐related ICE‐like caspase (Drice) and Death caspase‐1 (Dcp‐1). Under normal conditions, Dronc is targeted for degradation by the E3 ubiquitin ligase Death‐associated inhibitor of apoptosis 1 (Diap1). Pro‐apoptotic proteins Reaper, Hid, Grim (RHG) can then antagonise Diap1 to stimulate apoptosis [96].

To understand how death regulates regeneration following injury, studies in *Drosophila* have taken the approach to inhibit apoptosis execution through the expression of the baculoviral protein p35, which blocks Drice and Dcp‐1 but allows Dronc to remain active. This generates the so‐called undead cells, which receive pro‐apoptotic stimulation and are fated for death through Dronc activation, yet are not eliminated. Such models have revealed that components of the apoptotic machinery, particularly Dronc [95], can exert nonapoptotic inflammatory functions. In the context of undead cells and injury, both autonomous and nonautonomous increases in proliferative behaviours have been observed [93, 94, 95], and this process is thus designated apoptosis‐induced proliferation (AiP). This proproliferative stimulatory behaviour of undead cells is driven by a reactive oxygen species (ROS) increase by dual oxidase (Duox), which together with the JNK inflammatory pathway seems to drive the induction of senescence‐associated secretory phenotype (SASP), which includes mitogens such as Wingless (wg) and Decapentaplegic (dpp) [93, 94, 97, 98].

Resistance to cell death is a hallmark of cancer, allowing tumour cells not only to survive but also to chronically exploit proproliferative nonapoptotic caspase activity without dying. Mutations that increase mitogen‐activated protein kinase (MAPK) signalling, such as oncogenic *Ras* (Ras^V12^), promote the resistance to apoptosis [99]. Indeed, *Ras* ^*V12*^ driven tumours coupled with polarity loss have been shown to behave like undead cells that exploit the AiP response. Inhibition of the AiP executors, the effector caspase Dronc and ROS, strongly reduces tumour growth [100]. Furthermore, DNA damage events generated by ionising radiation or chromosomal instability, common features of tumour initiation and evolution, can potently promote undead cell behaviour and AiP. Cells experiencing such DNA insults and simultaneously inhibited from undergoing apoptosis, acquire sustained JNK activity, elevated ROS, and a senescence state characterised by cell cycle arrest and SASP [101, 102, 103, 104, 105].

Besides promoting their own growth through exploiting AiP, similar to the behaviour of undead cells, tumour cells can also stimulate the proliferation of neighbouring untransformed cells. Tumours generated with activated Raf and JNK signalling induce extensive nonautonomous hyperplasia in adjacent tissues [66]. Likewise, *Ras* ^*V12*^‐driven tumours experiencing stress, including DNA damage [72] or mitochondrial dysfunction [73, 74], induce nonautonomous proliferation through elevated JNK activity, increased ROS, DNA damage and p53 activation (Fig. 2A). Similar to undead cells, these stressed tumour cells also have a SASP phenotype, which includes the expression of the cytokine Unpaired (upd)/IL‐6, which activates JAK–STAT signalling in surrounding healthy cells [73, 74].

The endosomal sorting complexes required for transport (ESCRT) is frequently altered in cancer [106] and LOF of genes of this complex have also been found to drive potent nonautonomous hyperplasia. Mutations in components of the ESCRT complex, such as *vps25* and *erupted* (*tumour susceptibility gene 101‐TSG101*), disrupt endosomal sorting and result in aberrant Notch receptor accumulation and sustained Notch signalling [68, 69, 70]. This similarly activates the secretion of Upd, triggering JAK–STAT signalling in neighbouring cells, which causes pronounced nonautonomous tissue overgrowth [68, 69, 70]. Coincidentally, disruption of Rab5 GTPase, essential for early endosome formation, similarly promotes nonautonomous proliferation [71].

Overall, these studies highlight how tumours can hijack regenerative compensatory proliferative programmes in the microenvironment to sustain their growth and induce abnormal hyperplasia in neighbouring tissues.

### 4.2 Interactions between tumour subclones

#### 4.2.1 Subclonal interactions enhance tumour aggressiveness

During tumour evolution, transformed cells progressively accumulate distinct mutations, giving rise to subclones, each with a unique genetic lesion identity. In patients, mutational heterogeneity is associated with a worse prognosis [107, 108, 109, 110]. One significant issue arising from tumour heterogeneity is the potential emergence of subclones resistant to the therapy in use, which can ultimately drive relapse. However, it was unclear whether the coexistence of different tumour subclones can be responsible for driving tumourigenesis.

Pioneering studies in *Drosophila* have demonstrated that cooperative interactions between tumour subclones can indeed promote malignant behaviour. *Ras* ^*V12*^ clones generated in the EAD form small benign tumours, but they do not have the capacity for sustained growth or invasion to the nearby CNS. Strikingly, generating *scrib* mutant cells neighbouring *Ras* ^*V12*^ tumours induced massive overgrowth of the *Ras* ^*V12*^ tumours and invasion to the CNS [77] (Fig. 2B). Similar to the inflammatory behaviours discussed in the previous section, this cooperative overgrowth is mediated by JNK stress signalling in the *scrib* mutant subclones, which induces the secretion of Upd cytokines and stimulates JAK/STAT signalling in adjacent *Ras* ^*V12*^ tumour cells [77].

A recent study has identified cooperative interactions between two other subclonal mutational identities. Individually, neither *Ras* ^*V12*^ nor *Src* oncogene overexpressing (*Src* ^*OE*^) subclones exhibit invasive behaviour. However, when positioned adjacently, they reciprocally stimulate each other's invasive potential [78] (Fig. 2B). Mechanistically, *Ras* ^*V12*^ clones signal to *Src* ^*OE*^ clones via Delta‐Notch signalling, which induces the expression of the EMT‐associated ZEB‐like transcription factor *Zinc finger homeodomain 1* (zfh1), required for the invasion of *Src* ^*OE*^ cells into the CNS. In turn, Notch signalling is required for the induction and secretion of the Upd cytokine from *Src* ^*OE*^ clones, and disruption of this signalling axis prevents the reciprocal induction of *Ras* ^*V12*^ cell invasion [78]. These findings reveal that distinct oncogenic subclones can cooperate through spatially confined signalling interactions to enhance overall tumour progression. Interestingly, however, while *Ras* ^*V12*^ and *scrib* ^*−*^ mutations within the same cell trigger strong cooperativity, autonomous co‐mutation for *Ras* ^*V12*^ and *Src* ^*OE*^ does not induce invasion, in contrast with the interclonal setting *Ras* ^*V12*^//*Src* ^*OE*^ [78]. This suggests that there is an incompatibility between *Ras* ^*V12*^ and *Src* ^*OE*^ occurring within the same cell, and that the *Src* ^*OE*^ insult needs to be outsourced to a neighbouring population for cooperativity.

These two studies reveal that neighbouring subclones can cooperate to drive tumour progression and provide insight into the underlying molecular mechanisms. However, whether tumour subclones can also compete and thereby influence the dynamics of tumour evolution remains largely understudied. One study, however, has shown that the sequential accumulation of mutations within a single tumour subclone can shift the competitive balance between subclones [79]. Double‐mutant cells for *Ras* ^*V12*^ and *scrib* loss can outcompete neighbouring single‐mutant *scrib* cells (Fig. 2B). Mechanistically, *Ras* ^*V12*^, *scrib* ^*−*^ have increased ROS and mechanistic target of rapamycin (mTOR) activation when neighboured by *scrib* ^*−*^ cells, which leads to their death and engulfment by *Ras* ^*V12*^, *scrib* ^*−*^ cells [79].

Altogether, these findings highlight that both cooperative and competitive interactions between tumour subclones can shape tumour behaviour and potentially affect cancer progression.

### 4.3 Local interactions between tumour cells and the immune system

#### 4.3.1 The dual identity of haemocytes: from wound healers to tumour promoters

Although lacking an adaptive immune system, *Drosophila melanogaster* serves as a powerful model for studying the role of innate immunity *in vivo*. As in mammals, injury in *Drosophila* activates cellular immune responses. Macrophage‐like plasmatocytes are mobilised and migrate towards wound sites [111, 112]. Calcium flashes triggered upon tissue damage stimulate Duox activity, leading to the production of H_2_O_2_ and ROS, which in turn promote plasmatocyte recruitment to the wound [98, 113]. Among other factors required for stimulation of this immune cellular response [114], basement membrane disruption has been identified as key signal that guides haemocytes to sites of injury [115, 116]. At the site of damage, haemocytes have been shown to be required for the regenerative AiP response via stimulation of the JNK inflammatory pathway, likely through the secretion of Eiger (Egr)/Tumour Necrosis Factor alpha (TNFα) [98].

Tumours, which are often regarded as chronic wounds, similarly trigger a cellular immune response in *Drosophila*, which draws many parallels with the wound‐healing scenario. Larvae bearing tumours with mutations in epithelial polarity genes have been reported to exhibit increased haemocyte proliferation and, consequently, elevated numbers of circulating haemocytes [115, 117]. This expansion appears to be regulated by JAK–STAT signalling [115] and PDGF‐ and VEGF‐receptor‐related (Pvr) signalling [117], likely in response to tumour‐secreted ligands released into the circulation. Furthermore, there are increased numbers of haemocytes associated with tumours compared to wild‐type tissue [118]. Similar to the wound‐induced response, detoxification of ROS produced by tumours suppresses the accumulation of tumour‐associated haemocytes [98] (Fig. 2C). In addition, tumours often disrupt the basement membrane to facilitate invasion through matrix metalloproteinases (Mmps). Preventing the activity of tumour‐derived Mmps through overexpression of tissue inhibitor of metalloproteinase (TIMPs) strongly reduces the number of tumour‐associated haemocytes [119].

Several studies have investigated the function of haemocytes during tumourigenesis through the targeted perturbation of this cell population, revealing context‐dependent roles. In models where tumours are induced by single‐hit mutations in epithelial polarity genes, ablation of haemocytes via expression of the pro‐apoptotic gene *hid* leads to increased tumour size [115, 117]. Conversely, increasing the number of circulating haemocytes by promoting their uncontrolled proliferation through *Ras* ^*V12*^ results in smaller tumours [117]. Similarly, in a *Notch* hyperactivation allograft tumour model, depletion of haemocytes resulted in faster tumour growth over time [120]. These results suggest that haemocytes play a tumour‐suppressive role in this context. This suppressive effect appears to be mediated, at least in part, by the ability of haemocytes to induce apoptosis in polarity‐deficient tumour cells via the release of Egr [117] (Fig. 2C).

However, in the double mutant *Ras* ^*V12*^, *scrib* ^*−*^ model, haemocytes have been proposed to play a protumorigenic role (Fig. 2C). Strikingly, *Ras* ^*V12*^, *scrib* ^*−*^ tumours induce a stronger recruitment of haemocytes than single mutated tumours [118]. The JNK signalling pathway has been described as a double‐edged sword. While it can induce the death of polarity‐deficient clones, it instead promotes the growth and invasion of *Ras* ^*V12*^, *scrib* ^*−*^ tumours [118]. The ability of oncogenic Ras to override the antitumourigenic effects of JNK is likely due to its capacity to inhibit Hid activity [99, 121]. In this context, haemocytes are thought to contribute to tumour progression by promoting JNK activation in *Ras* ^*V12*^, *scrib* ^*−*^ tumour cells through the release of Egr [118].

Furthermore, haemocytes are also known to engulf and clear cellular debris and apoptotic cells from tissues [119, 122, 123, 124]. Although this behaviour might initially be considered as antitumourigenic by potentially preventing accumulation of inflammatory damage‐associated molecular patterns (DAMPs), recent work has shown that, in *Ras* ^*V12*^, *scrib* ^*−*^ tumours, this haemocyte activity actually promotes tumour growth (Fig. 2C). Downregulation in haemocytes of the engulfment receptor *draper* and other players involved in phagocytosis, including SH2 ankyrin repeat kinase (Shark) and Ced‐6, results in smaller *Ras* ^*V12*^, *scrib* ^*−*^ tumours. Similarly, affecting the exposure of phosphatidylserine (PS) in tumours, an eat‐me signal recognised by draper [125], resulted in decreased tumour growth [119]. Mechanistically, phagocytosis of tumour apoptotic cells leads to the increased haemocyte expression of *upd3*, which further amplifies *upd1/2/3* expression and JAK/STAT signalling in *Ras* ^*V12*^, *scrib* ^*−*^ tumours to stimulate proliferation [119].

Besides cellular immunity, humoral factors, such as AMPs, also shape tumourigenesis in *Drosophila*. Polarity‐deficient tumours induce systemic expression of the AMP Defensin across multiple organs, including the gut, fat body and trachea, and host‐derived Defensin constrains tumour growth by inducing cell death in transformed cells [126]. In the haematopoietic tumour model of *multi sex combs* (mxc) mutants, several AMPs, including *Drosomycin*, *Defensin*, *Diptericin* and *AttacinA*, are upregulated, and ectopic expression of these AMPs in the fat body is sufficient to reduce haematopoietic tumour burden [127].

Together, these studies demonstrate the context‐dependent roles of haemocytes in shaping tumour progression, acting either as tumour suppressors or as promoters depending on the genetic insults of the cancer cells.

## 5 Macroenvironmental interactions

Beyond local cellular interactions, tumours also engage in long‐range communication with distant host tissues. These interactions are mediated by tumour‐secreted factors that disrupt systemic homeostasis (Table 2), giving rise to a spectrum of physiological aberrations collectively referred to as paraneoplastic syndromes. Importantly, these tumour‐induced alterations can profoundly influence tumour progression and host survival. Thus, uncovering the mechanisms by which tumours co‐opt systemic physiology is of critical importance. In this section, we highlight the diverse macroenvironmental phenomena uncovered through studies in *Drosophila melanogaster*, which provide fundamental insights into how tumours influence, and are influenced by, the broader host organism.

**Table 2:** Tumour‐secreted proteins, target organs, and remote effects.

Tumour‐secreted factor	Target organ/tissue	Effect	Human orthologue	Ref.
**Cachexia**				
ImpL2	Fat body, muscle, reproductive organs	Antagonises insulin signalling → reduced PI3K/pAKT activity, increased Foxo targets, hyperglycemia, autophagy, mitochondrial fusion, lipid β‐oxidation → tissue wasting	*IGFBP* family	[128, 129, 130]
Pvf1	Fat body, muscle, corpora cardiaca	Activates Pvr/MAPK signalling → lipid loss, hyperglycemia, muscle defects, impaired mobility; in CC increases AKH → further lipid loss and hyperglycemia	*PDGF/VEGF* family	[131, 132]
Upd3	Muscle, fat body	Activates JAK–STAT → mitochondrial defects, reduced ATP, mobility loss; antagonises insulin signalling by inducing ImpL2 in muscle; drives gluconeogenic enzyme expression (Pepck1, Pdk) in fat body → hyperglycemia, mobility loss.	*IL‐6*	[133, 134]
Mmp1/2	Fat body	Remodel ECM, increase gbb availability → elevated TGFβ signalling → excessive ECM deposition, impaired muscle homeostasis, muscle wasting	*MMP* family	[135, 136]
**Anorexia**				
Dilp8	CNS (Lgr3^+^ neurons)	Activates Lgr3 → alters neuropeptides (↑NUCB1, ↓NPF) → appetite suppression (anorexia)	Relaxin family peptides	[137]
Upd3 + ImpL2	CNS (NPF neurons)	Reduce orexigenic NPF signalling → anorexia	*IL‐6* + *IGFBP* family	[138]
**Coagulopathy**				
Fon, Hml, Tg	Hemolymph/clotting system	Induce hypercoagulability → crystal cell depletion, hypocoagulability, impaired melanization, reduced survival	Functional analogues to fibrin	[139]
**Blood–brain barrier disruption**				
Upd	Blood–brain barrier (subperineurial glia)	Activate JAK–STAT → increased permeability, disrupted septate junctions, reduced survival	*IL‐6*	[140]
**Renal dysfunction & fluid imbalance**				
Upd	Malpighian tubule stellate cells	Activate JAK–STAT in stellate cells →? → stem cell proliferation in ureter → lumen obstruction, hypervolemia	*IL‐6*	[141]
Pvf1	Malpighian tubule principal cells	Activate Pvr/JNK/Jra signalling → stone formation → hypervolemia	*PDGF/VEGF* family	[142]
ITP_F_	Malpighian tubule stellate cells	Activates TkR99D GPCR → NOS‐cGMP signalling → reduced fluid secretion → hypervolemia	?	[143]

### 5.1 Cancer cachexia

#### 5.1.1 Metabolic sabotage: insulin signalling disruption and the breakdown of host tissues

**Fig. 3:**
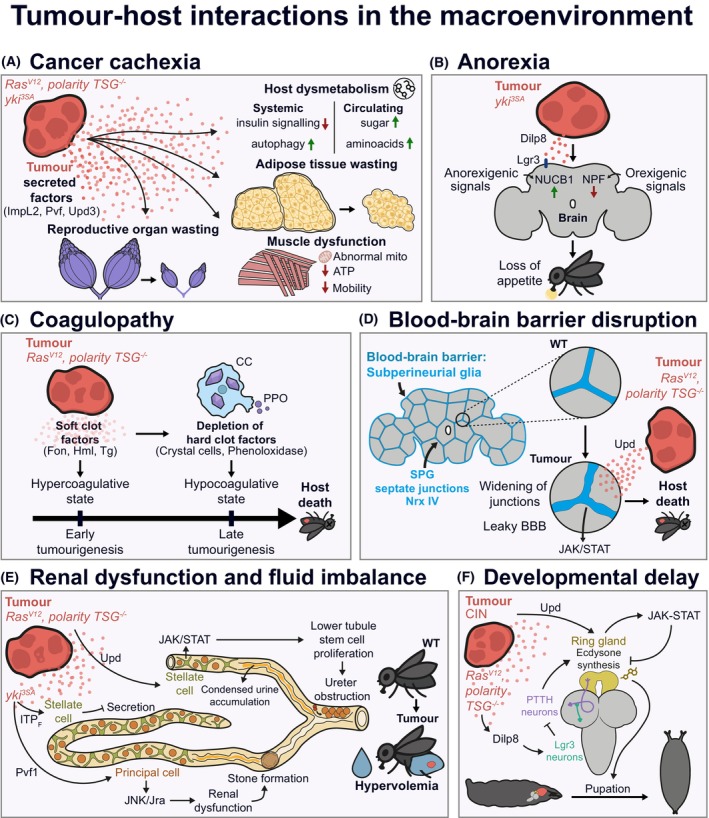
Tumour‐secreted factors drive the systemic disruption of host physiology. Illustrations depicting key macroenvironmental tumour–host interactions identified in *Drosophila* models. (A) Systemic wasting of host tissues (cachexia) is driven by metabolic dysregulation and inflammatory factors. (B) Tumours can induce loss of appetite (anorexia) by altering peptidergic signals regulating feeding behaviour. (C) Secretion of coagulogens by tumours promotes a hypercoagulable state that later transitions into hypocoagulation due to depletion of hard clotting factors, contributing to host death. (D) Inflammation in subperineurial glia (SPG) triggered by tumour‐derived cytokines increases blood–brain barrier (BBB) leakiness and reduces survival. (E) Tumour‐bearing animals can develop renal dysfunction through multiple mechanisms: inflammatory signals in stellate cells induce paracrine proliferation of lower tubule stem cells, leading to ureter blockage; tumour‐derived antidiuretic hormone (ITP_F_) blocks fluid secretion; and tumour‐derived Pvf1 activates JNK stress signalling in principal cells, promoting stone formation. These effects lead to excessive fluid accumulation and hypervolemia in the host. (F) Tumour‐secreted factors disrupt endocrine signalling and delay metamorphosis. Tumours upregulate Dilp8, which acts on Lgr3‐expressing CNS neurons to inhibit PTTH neurons innervating the ring gland. Reduced PTTH input suppresses ecdysone synthesis, thereby postponing pupation. In parallel, tumour‐derived Upd activates JAK/STAT signalling in the ring gland and further reduces ecdysone production. Chromosomal instability (CIN) tumour model is driven by loss of *bub3* and p35‐mediated death inhibition.

Cancer cachexia is one of the most prevalent and life‐threatening paraneoplastic syndromes, contributing to an estimated 20% of cancer‐related deaths [144]. It is primarily characterised by the progressive loss of skeletal muscle and adipose tissue, and notably, it cannot be fully reversed by nutritional supplementation [145], distinguishing it from starvation‐induced wasting. Strikingly, several larval and adult *Drosophila* tumour models have been shown to recapitulate key features of cachexia [128, 129, 146, 147] (Fig. 3A). In adult flies, fat tissue is progressively depleted, while lipid droplets become enlarged, along with mobilisation of stored lipid content [128, 129]. Muscles display disrupted mitochondrial morphology and reduced ATP levels, which are associated with impaired mobility [128, 129]. The reproductive organs of both females and males also undergo atrophy, with ovary apoptosis and degeneration being particularly striking [128, 129].

Systemic tissues of tumour‐bearing animals exhibit reduced phosphatidylinositol 3‐kinase (PI3K) activity, increased expression of Foxo target genes, and lowered pAKT levels. This suggests that a shift from anabolism to catabolism in systemic tissues, driven by disrupted insulin signalling, underlies the wasting phenotype [128, 129]. The haemolymph of tumour‐bearing flies also shows hyperglycaemia, likely indicating an inability to uptake circulating sugar due to impaired insulin signalling. A key driver of this disruption is *ImpL2*, the *Drosophila* homologue of insulin growth factor‐binding proteins (IGFBPs), which is secreted by tumours and antagonises insulin signalling. ImpL2 has been shown to be induced in tumours by the pro‐tumorigenic Hypoxia‐inducible factor 1 (HIF‐1α)/Sima [148]. Remarkably, downregulation of tumour‐derived ImpL2 restores pAKT levels, preserves tissue integrity, improves mobility and reduces circulating sugar levels [128, 129].

A central question is whether the loss of insulin signalling causes tissue wasting by passively halting growth or by actively inducing catabolic pathways. Autophagy, which is commonly activated upon reduced insulin signalling [149], has been shown to be induced in peripheral tissues of tumour‐bearing animals in response to tumour‐secreted ImpL2 [130, 146, 150]. Autophagy is a cellular process in which the formation of a double membrane structure encapsulates cytosolic cargoes for degradation and nutrient recycling upon fusion with the lysosome. Strikingly, genetic disruption of autophagy initiation machinery markedly prevents muscle wasting and impairs tumour growth [146]. In addition to autophagy, a distinct catabolic pathway has also been implicated in driving muscle wasting. ImpL2 induces mitochondrial fusion via Foxo and promotes the depletion of muscle lipid stores through fatty acid β‐oxidation, which drives wasting. Notably, dietary fat supplementation alleviates muscle wasting in this context [130].

Furthermore, one study has identified how paradoxically tumours sustain their own growth while promoting systemic tissue wasting through ImpL2‐mediated suppression of insulin signalling. Tumour models that express high levels of ImpL2 were found to depend on the expression of the Wnt ligand *wg* for continued growth. In the absence of *wg*, ImpL2 completely abolishes tumour growth [151]. Although the precise mechanism remains unclear, previous studies have shown that, in response to a high‐fat diet, tumours upregulate *wg*, which in turn increases expression of the insulin receptor (InR) [152]. This compensatory mechanism may explain the requirement for *wg* in ImpL2‐expressing tumours to increase local insulin sensitivity. Interestingly, *wg* expression in peripheral tissues of tumour‐bearing animals was also found to protect against cachexia [151].

#### 5.1.2 Tumour‐derived factors promoting cachexia

Beyond ImpL2, several other tumour‐derived factors have been found in flies to promote cachexia. Tumours generated in the ISCs of the gut through overexpression of oncogenic *yki* (yki^3SA^) upregulate *PDGF‐ and VEGF‐related factor 1* (Pvf1), a ligand that signals through the Pvr receptor [131]. Pvf1 was found to be required for inducing key cachexia‐like phenotypes, including muscle defects, impaired mobility, reduced lipid stores and elevated circulating sugar levels [131]. These effects are mediated by activation of the MAPK pathway in peripheral tissues, such as the fat body and muscle, through Pvr signalling. Notably, inhibition of MAPK signalling specifically in host tissues suppresses these wasting phenotypes [131].

In addition to its direct action in peripheral organs, Pvf1 was recently shown to act in neuroendocrine tissue, where it activates Pvr signalling in the CC to elevate AKH levels. Inhibition of Pvr signalling in the CC or deletion of *Akh* was sufficient to prevent tumour‐induced lipid loss and hyperglycemia in *yki* ^*3SA*^ tumour‐bearing animals [132]. Importantly, this Pvf1/Pvr/MAPK axis operates independently of ImpL2, highlighting the existence of distinct tumour–host signalling pathways capable of driving systemic wasting [131].

Another tumour‐secreted factor implicated in cachexia is the cytokine Upd3, a ligand of the JAK–STAT signalling pathway [133]. In *yki* ^*3SA*^‐driven gut tumours, *upd3* is strongly upregulated in an autonomous manner, leading to systemic activation of JAK–STAT signalling in host tissues. Disruption of *upd3* expression specifically in tumour cells is sufficient to prevent all major wasting phenotypes [133]. Ectopic activation of JAK–STAT signalling in the muscle is sufficient to impair mitochondria, reduce energy production, and compromise mobility [133]. Additionally, JAK–STAT signalling was found to antagonise insulin pathway activity, suggesting that Upd3 may act in concert with ImpL2. Supporting this, tumour‐derived Upd3 can induce the expression of ImpL2 in muscle tissue, revealing a mechanism by which inflammatory cues from the tumour stimulate host tissues to promote their own wasting via ImpL2 upregulation [133].

Moreover, recent studies using a *Drosophila* larval tumour model driven by *Ras* ^*V12*^ in combination with polarity gene loss revealed that tumour‐secreted Mmps contribute to cachexia by disrupting cell adhesion and promoting aberrant extracellular matrix (ECM) deposition in the fat body [135, 136]. Mechanistically, Mmps increase the availability of tumour‐derived ligand Glass bottom boat (gbb), which in turn leads to elevated TGFβ signalling in the fat body. This aberrant TGFβ activation further drives excessive ECM deposition within the fat body [135, 136]. Strikingly, these studies showed that such ECM dysregulation contributes to muscle wasting, likely because the ECM, which is normally produced by the fat body, is not properly delivered to the muscle, thereby impairing muscle homeostasis [135, 136].

Collectively, these *Drosophila* studies illustrate that tumour‐induced cachexia is driven by multiple secreted factors that act through both independent and converging mechanisms to promote tissue wasting.

#### 5.1.3 Stealing from the host to feed the tumour: nutrient mobilisation and tumour uptake

While cachexia drives extensive breakdown of host tissues, a central question is if and how tumours exploit this systemic metabolic rewiring to their advantage. Rather than simply disrupting host tissue homeostasis, evidence suggests that cachectic processes play a tumour‐supportive role by mobilising stored nutrients from peripheral organs to sustain cancer progression.

Stable Carbon isotope tracing experiments have shown that while *Ras* ^*V12*^, *scrib* ^*−*^ tumours initially accumulate biomass from nutrients sourced from food, during later stages of tumourigenesis, tumour growth is predominantly fuelled by nutrients derived from host tissues [146, 153]. Several amino acids increase in circulation, likely due to enhanced catabolism and release from wasting host tissues. Notably, autophagy, which is induced in peripheral tissues during cachexia, has been directly implicated in the elevation of specific amino acids in the hemolymph of cachectic *Ras* ^*V12*^, *scrib* ^*−*^ animals [146].

In addition to amino acids, circulating sugar levels rise sharply during cachexia. Glycogen stores in the fat body become depleted at late tumour stages, and this breakdown is also partially driven by autophagy [146], likely contributing to the observed hyperglycaemia. Moreover, a recent study in cachectic *yki* ^*3SA*^ animals revealed increased expression in the fat body of two key enzymes involved in gluconeogenesis: *Phosphoenolpyruvate carboxykinase 1* (Pepck1) and *Pyruvate dehydrogenase kinase* (Pdk) [134]. Fat body‐specific downregulation of *Pepck1* and *Pdk* in tumour‐bearing animals led to reduced levels of the circulating sugar trehalose. This upregulation of gluconeogenic enzymes and trehalose production was shown to be driven by the cytokine *upd3*, and JAK–STAT signalling in the fat body. Importantly, inhibition of *Pdk* in the fat body improved both mobility and survival of tumour‐bearing animals [134].

How tumours take up nutrients from the environment released from the host during cachexia and utilise them to fuel malignancy remains an understudied question. Nevertheless, several studies in *Drosophila* have identified nutrient transporters that can uptake metabolites from the environment and are essential for tumourigenesis. These include the amino acid transporters *slimfast* (slif) [150], the heterodimeric complex *CD98 heavy chain* (CD98hc) and light chains *Juvenile hormone Inducible‐21* (JhI‐21) and *minidiscs* (mnd) [154, 155], and *Pathetic* (Path) [156]. In neural stem cell‐derived tumours, the myc oncogene elevates *CD98hc* expression, driving increased TOR signalling, likely via enhanced uptake of amino acids that activate the TOR kinase [154]. Similarly, in *Ras* ^*V12*^, *scrib* ^*−*^ tumours, JNK and yki signalling promote the expression of *JhI‐21*, supporting tumour growth through increased TOR activity [155].

Overall, these studies illustrate how tumours can induce systemic tissue wasting and harvest host nutrients to sustain tumour growth.

### 5.2 Anorexia

#### 5.2.1 Appetite dysregulation in *Drosophila* tumour models

In addition to cachexia‐induced wasting, anorexic behaviour (defined by a persistent lack of appetite) is also frequently observed in cancer patients and contributes to progressive weight loss and tissue atrophy. Similar to cachexia, anorexia is thought to be driven by factors secreted into circulation [6, 157]. Feeding behaviour is regulated at multiple levels, integrating both external sensory cues and internal physiological signals, including circulating hormones/neuropeptides that can either promote food intake (orexigenic) or suppress it (anorexigenic) [158].

Two recent studies have used *Drosophila* to identify how tumour‐derived factors promote anorexia via the modulation of neuronal circuits associated with feeding [137, 138]. Animals bearing *yki* ^*3SA*^ tumours in the retina exhibit a pronounced reduction in food intake compared to controls. Among the various upregulated tumour‐secreted factors, the *Drosophila insulin‐like peptide 8* (Dilp8) was identified as a key mediator of the anorexic behaviour [137] (Fig. 3B). In *yki* ^*3SA*^ tumour‐bearing flies, the expression of the Dilp8 receptor, *Leucine‐rich repeat‐containing G protein‐coupled receptor 3* (Lgr3), is upregulated in the brain, and mutation of *Lgr3* increases food intake, supporting the idea that tumour‐derived Dilp8 acts via Lgr3^+^ neurons to suppress feeding [137]. Within the brain, activation of the Dilp8‐Lgr3 signalling axis alters the expression of neuropeptides that regulate appetite, leading to upregulation of the anorexigenic factor *nucleobinding 1* (NUCB1) and downregulation of the orexigenic *neuropeptide F* (NPF) [137].

In a separate study, flies with *yki* ^*3SA*^ tumours in the gut were found similar to have reduced feeding behaviour [138]. From the different tumour‐secreted factors, the combined action of the inflammatory cytokine Upd3 and the ImpL2 was found to be sufficient to induce the anorectic behaviour. In similarity to the previous study, the combined elevated levels of Upd3 and ImpL2 were found to trigger a depletion of orexigenic NPF, which contributed to weight loss [138]. Interestingly, tumour‐induced appetite loss was nutrient‐specific. Flies reduced their intake of yeast (a major protein source) and essential amino acids, but not sugar or fat. This protein‐specific anorexia was attributed to Upd3 and ImpL2 acting via reduced NPF signalling [138].

Together, these studies demonstrate that tumours can suppress appetite in *Drosophila* through secreted factors by altering neuropeptide signalling pathways in the CNS that regulate feeding.

### 5.3 Coagulopathy

#### 5.3.1 Tumours drive aberrant clotting response and host death

A commonly observed paraneoplastic syndrome in cancer patients is disseminated intravascular coagulation, which is characterised by an abnormal increase in systemic blood clotting [159]. Interestingly, coagulation abnormalities have also been reported in *Drosophila* cancer models [139, 160] (Fig. 3C). In flies, the clotting process occurs in two phases: an initial phase that involves the formation of a soft clot by local deposition of coagulogens, such as Fondue (fon), Hemolectin (Hml) and cross‐linking through Transglutaminase (Tg). This is followed by a second phase that leads to hard clot formation, driven by the melanisation response that is mediated by pro‐phenoloxidases (PPOs) released from lysed crystal cells [139, 161]. This clotting response is similar to the fibrin scabbing process in humans required to stop bleeding.

Strikingly, *Ras* ^*V12*^, *aPKC* ^*DN*^ tumours upregulate several members of the coagulation pathway, including *fon*, *Hml*, and Tg [139]. As a result, the haemolymph of tumour‐bearing animals enters a hypercoagulable state, which later progresses to a hypocoagulable phase in advanced disease. This transition appears to be driven by the tumour overproduction of coagulogens, which leads to the premature activation and depletion of crystal cells, resulting in reduced PPO activity and impaired melanisation [139].

Importantly, blocking this coagulopathy by depleting *fon* expression in tumour cells markedly improves the survival of tumour‐bearing flies [139]. Although the precise mechanism linking coagulopathy to reduced host viability remains to be elucidated, it is likely that excessive clotting, crystal cell exhaustion, and impaired injury repair contribute to systemic physiological decline. Notably, since crystal cells have recently been implicated in oxygen transport and respiratory function [12], their depletion may also lead to hypoxia, further compromising host viability.

### 5.4 Blood–brain barrier disruption

#### 5.4.1 Tumour‐derived inflammatory factors induce blood–brain barrier leakiness

The blood–brain barrier (BBB) constitutes a critical barrier between the CNS and host tissues that isolates the CNS from circulating pathogens and potentially harmful substances in the bloodstream, while allowing the selective transport of essential nutrients and molecules. In Drosophila, the BBB is formed by different glial cells and is functionally analogous to the endothelial BBB in vertebrates [162]. The subperineurial glia (SPG) envelop the CNS in a continuous epithelial layer and isolate it by forming tight septate junctions [162]. This barrier function by the BBB is vital to the host, as disruption of septate junction assembly leads to increased permeability and reduced survival [140, 163, 164].

Recent work in *Drosophila* has linked BBB integrity to tumourigenesis (Fig. 3D). Flies with *Ras* ^*V12*^, *aPKC* ^*−*^ tumours were found to have a compromised BBB, characterised by increased permeability and disorganised and widened septate junctions between SPG cells [140]. Similar to other paraneoplastic effects, this BBB disruption was also found to be mediated by tumour‐derived remote signals. Strikingly, depletion of *upd* ligands from the tumour, or the Upd receptor *domeless* (*dome*) specifically in SPG, suppresses the BBB permeability defect. Accordingly, SPG cells show high levels of JAK–STAT pathway activation in tumour‐bearing animals, indicating that tumour‐derived inflammatory signals can directly target the BBB, disrupting its integrity [140]. Interestingly, other inflammatory insults such as a high‐fat diet or bacterial infection also similarly disrupted BBB integrity, showing that the barrier function of SPG cells is highly sensitive to systemic inflammation [140].

Similar to what has been observed for the coagulopathy paraneoplastic syndrome, this tumour‐induced BBB defect also significantly contributes to increased host mortality. Depleting *Stat92E* or *dome* in SPG of flies with tumours increased their survival by 45.5% and 33.3%, respectively [140]. Interestingly, however, animals with constitutive JAK–STAT activation in the BBB, which causes increased permeability, and transplanted with tumours lacking Upd cytokines still exhibited a marked reduction in lifespan when compared with animals with only constitutive JAK–STAT activation in the BBB. This suggests that while JAK–STAT signalling is responsible for the BBB permeability defect, an additional unidentified tumour‐derived factor is driving host lethality [140].

### 5.5 Renal dysfunction and fluid imbalance

#### 5.5.1 Tumour‐secreted factors target renal cells to disrupt fluid homeostasis

Renal disease is frequently observed in cancer patients and contributes significantly to a poorer outcome [165, 166], yet the mechanisms underlying this pathology remain poorly understood. While renal impairment is commonly attributed to the nephrotoxic effects of cancer therapies, the possibility that tumours can directly disrupt renal function through systemic signals has received less attention.

A common feature observed across several fly cancer models is the development of hypervolemia, characterised by a bloated appearance due to excess fluid accumulation within the body [128, 129, 141] (Fig. 3E). In animals bearing tumours, this condition results in a 2‐fold increase in body liquid weight and up to a 12‐fold elevation of haemolymph volume [141]. Consistent with this fluid imbalance, the Malpighian tubules exhibit a significantly reduced secretory capacity in flies with tumours [141]. Recent studies have shown that tumours can impair renal function through at least three mechanistically distinct axes, each involving specific cellular targets and signalling pathways within the renal tissue.

In flies bearing *Ras* ^*V12*^ tumours coupled with polarity loss, the lower Malpighian tubules appear distended and filled with a cloudy, condensed urine‐like substance near the ureter, suggesting impaired fluid flow [141]. Instead of a clear open lumen, in flies with tumours the tubules are crowded with excess cells that seem to block the passage of urine [141]. The lower tubule normally contains quiescent stem cells that only proliferate upon damage. However, in contrast to wild‐type animals, tumour‐bearing flies exhibit a proliferative response in the lower tubule and ureter, where stem cells give rise to principal cell‐like progeny that fill the lumen [141]. Strikingly, eliminating the presence of these stem cells from the tubule prevented this proliferative response and hypervolemia. This proliferative response was found to be commanded by the tumour via stellate cells. Mechanistically, tumours secrete Upd cytokines, which stimulate JAK–STAT in stellate cells, which in turn seem to stimulate proliferation of stem cells likely via the epidermal growth factor receptor (EGFR) MAPK pathway [141]. Thus, tumours can promote renal dysplasia via inflammatory signals that seem to obstruct fluid flow through the ureter, resulting in systemic fluid accumulation.

In another study, flies with *yki* ^*3SA*^ tumours generated in the gut ISCs were found to develop crystal stones in the lower portion of the Malpighian tubules [142]. Kidney stones typically form through the precipitation of urinary solutes, most commonly uric acid and calcium oxalate. Consistently, *yki* ^*3SA*^ flies exhibit increased levels of uric acid, a by‐product of purine metabolism. Feeding flies a purine‐rich diet exacerbates stone formation. Furthermore, chemical dissolving of calcium oxalate leads to reduced stones, suggesting these are formed by both compounds [142]. Importantly, this also reduced the frequency of hypervolemia, which suggests that stone formation blocks urine passage. Several genes important for renal function and elimination of uric acid had altered expression in the Malpighian tubules of tumour‐bearing animals. Strikingly, tumours were found to induce these remote disruptions on kidney function through Pvf1/Pvr/JNK/Jra signalling. Depleting the Pvr receptor in principal cells restores uric acid levels, reduces stone formation, and alleviates hypervolemia [142].

Moreover, it was recently shown that tumours can promote hypervolemia by secreting an antidiuretic hormone into circulation [143]. Specifically, a part of the tumour cells produces isoform F of the *ion transport peptide* (ITP_F_), which induces fluid retention by suppressing secretion in the Malpighian tubules. Secreted ITP_F_ binds the G‐protein‐coupled receptor (GPCR), Tachykinin‐like receptor at 99D (TkR99D) on stellate cells, activating the nitric oxide synthase (NOS)—cyclic guanosine monophosphate (cGMP) signalling cascade and thereby reducing fluid release [143].

These studies identify how tumours can drive renal dysfunction via multiple secreted factors, each targeting specific cell types within the Malpighian tubules.

### 5.6 Developmental delay

#### 5.6.1 Tumour‐secreted factors cause endocrine disruption and slowed development

Endocrine complications are common among cancer patients and can lead to a wide range of systemic alterations [167, 168], reflecting the central role of hormones in coordinating physiology throughout the body. This issue is particularly pronounced in childhood cancers, as growth and developmental timing during this life stage depend heavily on tightly regulated endocrine signalling. However, beyond cases where tumours arise within or compress hormone‐producing organs, it remains difficult to determine the extent to which endocrine organ dysfunction arises from therapeutic toxicity versus tumour‐induced macroenvironmental effects. Work using *Drosophila* larval tumour models has provided key insights into how tumours can remotely provoke endocrine dysregulation and cause developmental delay (Fig. 3F).

Previous work has shown that developing larvae possess a robust mechanism for inter‐organ growth coordination that ensures that if one tissue experiences impaired or slowed growth, other organs transiently reduce their own growth rates to maintain body‐wide proportionality. This coordination is mediated by the secretion of the hormone Dilp8 from damaged or slow‐growing tissues, which acts systemically by binding the Lgr3 receptor in specific CNS neurons, which inhibit the PTTH neurons that innervate the ring gland and positively regulate ecdysone synthesis [169]. Dilp8‐Lgr3 signalling thus leads to suppressed ecdysone production and a delay of the onset of metamorphosis, thereby allowing time for regeneration and coordination of organ growth [170, 171, 172, 173, 174].

As chronic wounds, tumours in *Drosophila*, such as *Ras* ^*V12*^, *scrib* ^*−*^ and other models, have been found to strongly upregulate Dilp8, and thereby trigger developmental delay [172, 175, 176, 177]. This Dilp8 induction and the consequent slowing of development are regulated by JNK signalling and the Ets21c transcription factor within the tumour [175]. Also, Dilp8 is regulated by the Hippo pathway, another critical signalling cascade involved in tumourigenesis [176]. Moreover, clinical data have described a link between delayed puberty and inflammatory disease [178, 179]. Studies in flies have identified a Dilp8‐independent inflammatory axis through which tumours can impose developmental delay [180, 181]. Tumours upregulate the JAK/STAT activating cytokine Upd3, which acts systemically to activate JAK/STAT signalling in the prothoracic gland. This results in increased activity of the bantam microRNA, which negatively regulates ecdysone production [180, 181]. Additionally, the transcription factor Apontic has been shown to be induced by JAK/STAT in the prothoracic gland and to contribute to metamorphosis delay [181].

Taken together, these mechanistic studies in *Drosophila* demonstrate that developmental delay can be directly induced by tumour‐secreted factors that misregulate endocrine organs.

## 6 Microbiota‐tumour–host interactions

### 6.1 Bacterial dysbiosis, inflammation, and systemic consequences in *Drosophila* tumour models

Animal bodies house complex microbial communities, collectively referred to as the microbiota, which play integral roles in shaping physiology, immunity and disease progression [182]. In contrast to the mammalian gut, which can host around 1000 species of microbes, the *Drosophila* microbiome is comparatively simpler, comprising at most 20 species [183]. This simplicity offers the advantage of enabling the use of the fly as a reductionist model to uncover novel mechanisms of host–microbe interactions [184, 185, 186]. The gut microbiota of *Drosophila* is mostly composed of bacterial members of the phyla Firmicutes (family *Lactobacillaceae* and *Enterococcaceae*) and Proteobacteria (family *Acetobacteraceae* and *Enterobacteriaceae*) [185].

Several *Drosophila* cancer models have been described to suffer from microbiota dysbiosis, characterised by both increased bacterial load and changes in species diversity in the gut [187, 188, 189]. Dysbiosis is frequently accompanied by epithelial barrier dysfunction, and modulation of the JNK pathway and Mmps within gut tumours, which has been shown to promote barrier breakdown and to reciprocally influence bacterial load levels [187]. Studies using axenic conditions or antibiotic treatment have shown that the microbiota can stimulate local tumour growth in the gut [187, 190]. Bacteria in the gut have been shown to increase the levels of protumorigenic inflammatory genes, such as *upd3* and *kayak* (kay) [187]. Strikingly, bacteria can also further impair gut barrier function, suggesting that the microbiota and tumour cells can engage in a loop that drives both dysbiosis and tumourigenesis [187].

Specific bacteria have been directly linked to tumourigenesis in *Drosophila* models. The commensal *Lactobacillus brevis* has been shown to promote tumour growth, whereas the closely related fly gut resident *Lactobacillus plantarum* does not [190]. Interestingly, this tumour‐promoting effect of *L. brevis* persists even when dead bacteria or its cell wall are administered to flies, suggesting that the species‐specific effect is detected by the host through sensing of *L. brevis* cell wall components [190]. Moreover, oral infection of flies with the human pathogen *Pseudomonas aeruginosa* has been shown to promote the dissemination of *Ras* ^*V12*^ gut tumours through activation of the immune deficiency (Imd) pathway [191].

Besides directly modulating local tumour growth, bacterial dysbiosis has been shown to affect the host during cancer progression. In tumour‐bearing flies, loss of gut barrier integrity appears to permit the translocation of bacteria [189] and bacterial peptidoglycans [188] into the haemolymph, resulting in systemic activation of the Imd pathway. Affected tissues include the muscles, as well as renal structures such as the Malpighian tubules [188] and heart tube‐associated nephrocytes [189]. Treatment with antibiotics does not rescue cachexia phenotypes, such as expression cachectins (Upd3, Pvf1, and ImpL2), ovary wasting, muscle defects, and reduced mobility [188]. However, tumour‐bearing flies raised under axenic conditions display an extended lifespan [187, 188, 189]. Strikingly, blocking the Imd innate immunity pathway in either Malpighian tubules [188] or nephrocytes [189] results in an increase in lifespan. Imd activation in the Malpighian tubules drives uric acid accumulation which increases mortality [188]. Moreover, Imd activation in tumour‐bearing animals induces expression of the antimicrobial peptide Attacin‐D in the Malpighian tubules, causing renal damage and further reducing lifespan [192].

Together, these studies position the *Drosophila* microbiota as an active participant in cancer biology, influencing not only local tumour growth but also systemic host health.

## 7 Sexual dimorphism and tumour–host interactions

### 7.1 Hormonal and inflammatory cue differences between sexes shape tumour dimorphism

Cellular sexual identity is defined by a well‐characterised cascade in *Drosophila*, where the RNA‐binding protein Sex‐lethal (Sxl) in females produces the active splicing factor Transformer (Tra), leading to the sex‐specific splicing of the transcription factors Doublesex (Dsx) and Fruitless (Fru) [193]. Beyond directing reproductive system development, somatic sexual identity also determines several other dimorphisms, including traits in physiology, immunity, organ size, behaviour and cellular stress mechanisms [193]. Moreover, in similarity to humans, sex‐specific hormonal differences have been documented in flies, such as the production of the steroid hormone ecdysone by ovaries in mated females [194]. Clinically, it is well‐recognised that many diseases show sex‐biased prevalence or severity [195], yet the mechanistic basis of these differences remains poorly understood.

In this context, *Drosophila* studies have demonstrated that host sex can influence tumourigenesis. In adult females, mating triggers extensive gut remodelling, characterised by increased ISC proliferation and gut size, to support nutrient uptake for oogenesis [196]. This response is regulated by the somatic sexual identity of ISCs [197] and by ovarian ecdysone [194], which stimulates ISC proliferation. However, this reproductive adaptation carries a trade‐off: mated females are more susceptible to age‐dependent gut dysplasia and to tumour growth following *Notch* loss in ISCs [194]. Notably, dietary administration of ecdysone to virgin females or males similarly promotes tumourigenesis, highlighting how hormonal regulation can contribute to sex‐biased tumour susceptibility [194].

Sexual dimorphism in tumour behaviour has also been reported in *Drosophila* brain tumour models. In a larval model driven by loss of *l(3)mbt*, tumours exhibited marked sexual dimorphism, whereas those driven by loss of *brat* did not [198]. In the *l(3)mbt* model, male larvae developed tumours with a more aggressive growth pattern than females. Strikingly, when *l(3)mbt* larval brain tumours were allografted into adult hosts, male‐derived tumours killed the host significantly faster than female‐derived tumours [198], indicating that tumour‐intrinsic sexual identity can influence both growth dynamics and lethality. Proteomic analysis revealed that male *l(3)mbt* tumours displayed a biased expression of the chromatin reader *PHD finger protein 7* (Phf7). Loss of *Phf7* in the *l(3)mbt* background abolished the dimorphic tumour growth pattern and host killing, identifying *Phf7* as a key mediator of this sexual tumourigenic dimorphism [198].

More recently, host sex has also been shown to influence the nature of tumour–host interactions. For example, cell competition [199] and *myc*‐driven supercompetition [200] exhibit sexually dimorphic activity levels. Moreover, in a larval model in which overactivation of Notch signalling in the salivary gland drives overgrowth, female tumours were approximately twice bigger in size compared to male tumours [201]. This dimorphism was associated with elevated inflammatory JNK signalling and higher expression of the JAK–STAT ligand *upd2* in female tumours [201]. Mechanistically, these differences were triggered by female‐biased expression of the JNK pathway inducing ligand Egr in haemocytes. Disrupting this signalling axis, either by depleting pathway components in the tumour or in haemocytes, reduced the sex‐biased increase in tumour growth [201]. Importantly, this dimorphism was brought upon by the somatic sexual identity cascade of *Sxl* and *Tra* in female haemocytes [201]. Furthermore, Upd2 secretion from the tumour‐induced increased release of Dilp2 from the insulin‐producing cells (IPCs), thereby enhancing insulin signalling in female tumours and promoting their overgrowth [201].

Overall, these studies demonstrate that sex‐specific differences in tumour–host biology can profoundly influence tumourigenesis.

## 8 Conclusion

Studies in *Drosophila* have converted our understanding of tumour–host biology from hypothesis and correlative observations into mechanistic explanations at several scales. At the microenvironmental level, tumour development is shaped by interactions with adjacent homotypic untransformed cells and innate cellular immunity, as well as through cooperative and competitive interactions among tumour subclones. At the macroenvironment level, tumours secrete factors that act at a distance to dysregulate systemic homeostasis, which can lead to tissue wasting, anorectic behaviour, blood–brain barrier leakiness, renal dysfunction and dysregulated haemostasis. These systemic changes end up having critical consequences for disease outcome, such as through fuelling tumour progression, or disrupting host tissue health and promoting mortality. Furthermore, the microbiota and host sex have emerged as important determinants of tumour–host biology.

Despite the differences between flies and humans, studies consistently show that basic principles uncovered in flies are conserved in vertebrates. The identified mechanisms that regulate tumour–host biology commonly converge on conserved signalling nodes, such as ROS, JNK, JAK–STAT, PDGFR and insulin/AKH signalling, highlighting several druggable targets in host tissues to modulate cancer progression. Yet therapeutic targeting of untransformed host tissues remains largely unchartered in clinical practice. Work in flies indicates that host‐targeted manipulations can exert strong antitumour effects, suggesting that, alongside targeting the tumour itself, modulating host tissues could further restrain tumour growth and blunt paraneoplastic pathology to protect the host and improve survival.

Looking forward to understudied questions, *Drosophila* could provide a valuable platform to interrogate the effects of diet and ageing on tumour–host biology, and for evaluating how host tissues contribute to therapy resistance and tumour relapse. The genetic tractability and systemic complexity offered by fly models provide unique resolution into the genetic players and the directionality of inter‐compartment/organ interactions. Thus, it is only expected that work in *Drosophila* will continue to provide fundamental discoveries in our understanding of tumour–host biology.

## Conflict of interest

The authors declare no conflict of interest.

## Author contributions

J.T.‐R. and T.E.R. conceived and designed the review. J.T.‐R. conducted the literature search, drafted the manuscript, and prepared the figures. Both authors contributed to editing and approved the final manuscript.
